# Volatile Profile Characterization of Croatian Commercial Sparkling Wines

**DOI:** 10.3390/molecules25184349

**Published:** 2020-09-22

**Authors:** Ana-Marija Jagatić Korenika, Darko Preiner, Ivana Tomaz, Ana Jeromel

**Affiliations:** 1 Department of Viticulture and Enology, Faculty of Agriculture, University of Zagreb, Svetošimunska 25, 10000 Zagreb, Croatia; amjagatic@agr.hr (A.-M.J.K.); itomaz@agr.hr (I.T.); amajdak@agr.hr (A.J.); 2 Center of Excellence for Biodiversity and Molecular Plant Breeding, Svetošimunska 25, 10000 Zagreb, Croatia

**Keywords:** sparkling wines, volatile aroma compounds, Zagreb county, discriminant analysis

## Abstract

Commercial sparkling wine production represents a relatively low but important part of the Croatian wine production, especially in the Zagreb county. This study presents the results of volatile aroma compounds profile and organic acid composition of commercial sparkling wine samples from three vine-growing regions in Zagreb county. In total, 174 volatile aroma compounds were identified, separated between their chemical classes (aldehydes, higher alcohols, volatile phenols, terpenes, C13-norisoprenoids, lactones, esters, fatty acids, sulfur compounds, other compounds, other alcohols). Higher alcohols such as phenylethyl and isoamyl alcohol as well as 2-methyl-1-butanol, and esters such as diethyl succinate, ethyl hydrogensuccinate, and ethyl lactate had the strongest impact on the volatile compounds profile of Zagreb county sparkling wine. The presence of diethyl glutarate and diethyl malonate, compounds whose concentrations are influenced by yeast autolysis or caused by chemical esterification during the ageing process, was also noted. The influence of every single volatile aroma compound was evaluated by discriminant analysis using forward stepwise model. The volatile profiles of traditional sparkling wines from Croatia were presented for the first time. It is hoped the results will contribute to better understanding the quality potential and to evaluate possible differences on the bases of detected aroma concentrations and multivariate analysis.

## 1. Introduction

According to International Organisation of Vine and Wine (OIV), in 2018, world sparkling wine production reached 20 million hectoliters with an overall increase of +57% since 2002. In global sparkling wine production, almost half of the total volume produced comes from Italy (27%) and France (22%), followed by Germany (14%), Spain (11%), and USA (6%) [1]. For the past ten years, Croatia has also recorded apparent increase in sparkling wines production, with the Zagreb County as one of the leading wine-growing counties. According to the Croatian Agency for Agriculture and Food data, in 2017, 885.80 hL of sparkling wines were produced in that area with a continuous upward trend. In Croatia almost all sparkling wines are produced by the traditional method, where marked influence can be connected to grape variety. Pjenušac is a quality sparkling wine (Protected Geographical Indication) elaborated by the traditional method that is defined by an excess pressure higher than 3.5 bar, primarily connected with presence of carbon dioxide in solution that is kept at the temperature of 20 °C, and for which alcoholic concentration of the cuvées used for their production have at least 9% volume (Council Regulation (EC), 1308/2013). In Zagreb county, ‘‘Pjenušac’’ is mainly produced from Riesling and Chardonnay grape cultivars and Pinot Noir between the red ones. However, there is also a great diversity of other grape varieties as Manzoni, Portugizer, Muller Thurgau, as well as Kraljevina and Plavec žuti, presenting a quality that can obtain high quality natural sparkling wines with their own personality and sensory profile. However, no work dealing with the influence of these grape cultivars for sparkling wine production have been done. The quality of sparkling wines is mainly influenced by their aroma composition and properties of single aroma compound present [2,3,4]. The sparkling wines aroma composition is formed by the interaction of different factors, such as grape variety and its maturity level, the production technology, the primary and secondary methods of wine fermentation, type of yeast strain used, storage temperature, ageing period, and ingredients used for liqueur d’expedition, level of oxygen during the process of disgorging, type of closure used, and levels of SO_2_ as well as CO_2_ [5,6]. According to Kemp et al. [6] wines used in the dosage solutions can have strong impact on volatile compounds concentrations, more than concentration of added sugar. An increase of ethyl esters, such as diethyl succinate, alcohols, and some varietal aromas, such as TDN (1,1,6-trimethyl-1,2-dihydronapthalene) and vitispirane, connected with fermentation in the bottle was noted by several authors [7,8,9], while concentrations of acetic acid esters and fatty acids diminish because of their clevage to the yeast cell walls [7,9]. In the work by Muñoz-Redondo et al. [10] some ester compounds were pointed out as markers of the second fermentation. Aroma changes can be further modified during the ageing on lees, so, therefore, the ageing time can determine the volatile aroma profile present in the sparkling wine [7,11,12]. Loyaux et al. [13] studied the aroma composition changes during the champagne ageing period and detected a slow decrease of isoamyl butyrate and hexyl acetate levels, as well as nerolidol concentrations and an increase in benzaldehyde and vitispirane levels. Over a period of 16 years, the concentrations of benzaldehyde increased up to 4 mg L^−1^. Environmental factors such as terrain structure, agro- and amphelo-pedological characteristics, climate and viticulture practices used, often described as “terroir”, can also have strong influence on grape composition and wine quality. Geographical origin also has an significant role in the differentiation of wines, since it can indicate the resemblance among wines coming from the one specific vine-growing region and the main differences among the ones coming from several viticultural regions [14,15]. Wine aroma precursors, as well as most wine components, are mainly accumulated during the grape maturation process in the vineyard. They can form a recognizable pattern in the grapes that can enhances the unique nature and specific structure of wines. Studies by Goldner et al. [16] and Vilanova et al. [17] have demonstrated differences in the sensory characteristics of Malbec and Albariño wines from different geographic origins. Robinson et al. [18] noted that the volatile aroma profile of certain type of wine can have marked impact in obtaining a geographical designation by forming a product with characteristics of specific vine-growing area. Nowadays, Voce et al. [19] carried out a comprehensive mapping of sparkling wines samples according to their volatile aroma compounds from Trentodoc and Franciacorta to determine regional features among them. The main target of this research was to define the volatile compounds profile in a relatively significant number of sparkling wine samples from three vine-growing regions in Zagreb county and to evaluate possible differences on the bases of detected aroma concentrations and multivariate analysis. From our experience, this work represents the first definition of the chemical structure of Zagreb county sparkling wines.

## 2. Results and Discussion

### 2.1. Composition of Organic Acids

Organic acid profile of Croatian sparkling wines from three Zagreb county vine-growing regions is presented in Table 1. There was no significant difference observed among the sparkling wines in terms of main organic acids as well as pH values. The most abundant acid was tartaric with an average concentration between 2.06 and 2.35 g L^−1^, values similar to ones published by Focea et al., Caliari et al. and Gallardo-Chacón et al. [20,21,22], but much higher compared to results published by Sartor et al. [23]. Conversely, malic acid concentrations were relatively low when compared to literature data by Caliari et al. and Sartor and al. [21,23], and ranged between 0.81 and 1.31 g L^−1^. It is well known that, in the sparkling wine elaboration process, the grapes must be usually harvested before they are completely matured [24]. The lactic acid concentrations varied according to the region, and could be influenced by grape composition, as well as by yeast activity, formed from malic acid degradation. The concentration of succinic acid, formed during the fermentation process, was lower compared to data obtained in previous studies [21,23]. Citric acid was present in all sparkling wines samples contrary to the data obtained by Caliari et al. and Sartor and al. [21,23] where it was not detected, but in agreement with work by Gallardo-Chacón et al. [22].

### 2.2. Volatile Compounds

One hundred and seventy-one volatile compounds presented in sparkling wines from three different Zagreb county vine-growing regions were detected, quantified and classified into several chemical classes (aldehydes, higher alcohols, volatile phenols, terpenes, C13-norisoprenoids, lactones, esters, fatty acids, sulfur compounds, other compounds, other alcohols). In Table 2, the average values of main volatile compounds chemical classes are presented, showing a significant difference among vine-growing regions while individual volatile compounds are presented in Table 3. The most abundant class was higher alcohols group with the highest concentrations of isoamyl and phenylethyl alcohol as well as 2-methyl-1-butanol. Comparing these compounds among vine-growing regions shows that sparkling wines from Zelina had significantly the highest concentrations. Data from the work by Torrens et al., Caliari et al., and Torchio et al. [9,25,26] also showed that major aromatic compound was phenylethyl alcohol, with OAV > 1 having influence on the sweet, rose and honey aroma structure of sparkling wines. The concentrations of higher alcohols not exceeding the amount of 300 mg L^−1^ can positively influenced the formation of wine complexity [27] which was not the case in our samples. Besides above mentioned compounds *cis*-3-hexene-1-ol, had also an impact on “green grass” odour profile of Zagreb county sparkling wines, especially in some samples from Plešivica vine-growing region. Representatives of alcohols that are also characterized by “green” and “herbaceous” notes, such as *trans*-1-hexanol, and *cis*-2-hexene-1-ol, and *trans*-3-hexen-1-ol, which are mostly synthetized during the pre-fermentation wine production process, were detected, but in concentrations under the defined odour threshold value in all sparkling wines samples analysed. Yeast contact and storage time on lees during sparkling wine production might have been the reason for relatively higher concentrations of 1-hexanol that ranged from 1612 to 2948 µg L^−1^, concentrations that are in agreement with data reported by [28]. As it can be seen from the Table 3, the presence of 1-hexanol was significantly the highest in sparkling wines from Krašić while there were no marked differences between other two regions. According to Alexandre et al. [29] and Benucci et al. [30], esters are the main class of aroma compounds released by the degradation of yeast cells having low perception thresholds and so positively contributing to the aroma of fruit as well as floral-like aroma of sparkling wine. Significantly, the highest amount of esters was detected in sparkling wines from Zelina while there was no marked difference between the other two vine-growing regions. Also, it can be seen that total esters concentration was more or less similar or something higher when compared to the data published by Benucci et al. [30]. Among esters presented in the analysed sparkling wines, the most common were diethyl succinate, ethyl hydrogensuccinate, and ethyl lactate, which is comparable with the results published in the work by Benucci et al. [30], while the ones with the OAV > 1 were ethyl butanoate, hexanoate, octanoate, ethyl-2-methylbutanoate, ethyl-3-methylbutanoate, isoamyl acetate, and isoamyl lactate. Comparable results were achieved by Voce et al. [19] in Ribolla Gialla sparkling wines where esters had an important role in volatile profile structure. Ethyl decanoate (floral) and 2-phenylethyl acetate (scent of rose) were detected in all sparkling wines in concentrations under the odour threshold values but according to Genovese et al. [31] these compounds can show synergistic effect even at low concentrations. The concentrations of 2-phenylethyl acetate published by Torchio et al. [26] were comparable with our data (23.40 to 28.73 µg L^−1^). According to Torrens et al. [9] and Riu-Aumatell et al. [11] diethyl succinate and ethyl lactate are considered as “ageing esters” whose concentrations can increase in contact with yeast cells during the second fermentation. For the development of cava, diethyl succinate can be used as a marker, mainly connected with the period of cava storage in the cellar [11]. In Zagreb county sparkling wines its concentrations were between 3917.45 µg L^−1^ (Krašić) up to 7430.69 µg L^−1^ (Zelina) which is compared to Ribolla Gialla wines (2555 µg L^−1^) higher but compared to concentrations published by Martinez-Garcia et al. [32] ranging between 8900 µg L^−1^ and 15,000 µg L^−1^ much lower. Among other ester compounds detected in Zagreb county sparkling wines isobutyl lactate, ethyl-2-hydroxy-3-methylbutanoate, diethyl hydroxysuccinate and isobutyl lactate concentrations were significantly higher in Zelina vine-growing region wines while ethyl vanillate, phenyl acetate and ethyl-hydroxyhexanoate concentrations were significantly the highest in wines from Plešivica vine-growing region. In analysed sparkling wines, the presence of diethyl glutarate and diethyl malonate, compounds, whose concentrations are influenced by yeast autolysis or caused by chemical esterification during the ageing process, was detected. By the use of chemometric analysis, diethyl malonate was pointed out as one of the most important compounds having strong influence in the Chardonnay wines differentiation [33]. In the work by Carlin et al. [34], the above mentioned compounds were also reported with concentrations of diethyl glutarate (5.8–7.3 µg L^−1^) similar to our data (12.0–19.9 µg L^−1^). Sparkling wines from Krašić stood out with significantly the highest concentration of diethyl malonate, while there was no significant difference in diethyl glutarate concentrations among tested wines. Terpens as a large group of wine aroma compounds primarly characterized by floral aroma are translocated from the grape to the must during the pressing and settling process in free volatile form or bound to sugars. In wines, according to Bordiga et al. [35] the transformation of the monoterpenes is linked to corresponding pyranic and furanic oxides or reduction by yeast membrane incorporation and acetylation [36]. Changes in the aroma characteristics during wine maturation were investigated by Oliveira et al. [37], showing a marked increase in monoterpenic oxides and decrease in monoterpenic alcohols. In our research, the presence of *trans* and *cis* linalool oxide, furan as well as geranyl acetate was detected in sparkling wines from all three vine-growing regions. Tetrahydrolinalool was significantly the most abundant terpene in sparkling wines from Plešivica and Zelina vine-growing region, while the significantly highest concentrations of nerol, terpene-4-ol and geraniol were detected in wines from Zelina while terpendiol II and hotrienol was most common in wines from Krašić. According to Caliari et al. [25] the main monoterpenes presented in their work were hotrienol, geraniol, linalool, citronellol, *α*- terpineol and the oxide forms of linalool. In all sparkling wines, the odour threshold value of linalool was above one. This corresponds to our data pointing out linalool, geraniol and hotrienol as the compounds with OAV > 1. Among C13-norisoprenoids compounds detected *β*-damascenone and TDN were the most common with the significantly highest total concentration above odour detection threshold in sparkling wines from Zelina vine-growing region sparkling wines. TDN originate from carotenoid degradation that is influenced by the ageing process linked to acid-catalysed reactions [9]. Also, according to Marais et al. [38], the TDN levels were remarkably higher in grapes that had more sunlight during maturation than in grapes from shaded locations. So, there is a reason to point out a potential impact of pruning level as well as leaf removal on carotenoid levels [34]. In the work by Francioli et al. [7], TDN was pointed out as a compound that, together with diethyl succinate and vitispirane, can discriminate cavas aged >20 months. A significant difference was also detected in total fatty acids concentrations probably being connected with the different grapes origin, the concentration of lipid substances in the must and differences in winemaking conditions used [19]. The most representative fatty acids similar to data published by Voce et al. [19] were hexanoic, octanoic and decanoic acid with the highest concentrations detected in Plešivica vine-growing region sparkling wines and average concentrations higher than their odour detection threshold. These acids, depending on the concentration, can have negative role in the development of wine sensory profile [9,21], but Shinohara’s [39] data pointed out that, if the concentrations are from 4 to 10 mg L^−1^, they can positively influenced wine aroma, while if their concentrations are more than 20 mg L^−1^ they can negatively influence the organoleptic profile of wines what was not the case in our study. Among sulfur compounds detected in analysed sparkling wines 4-methylthio-1-butanol was previously pointed by Rapp [27] as a potential contributor to wine aroma. Its formation can be linked to the degradation of amino acids containing sulfur or as a process connected to sulfur pesticides degradation that are used in the grape protection [40]. Another identified sulfur compound was 3-methylthio-1-propanol which at the contrary has negative influence to aroma mainly due to odour descriptor defined as boiled potatoes, but in our work with no direct impact because of relatively high odour detection threshold. Influence of ageing and storage on lees on the concentration of some fermentative sulfur compounds during sparkling wine production was investigated by Fedrizzi et al. [41], showing significant increments for 4-methylthio-1-butanol as well as 3-methylthio-1-propanol. This result supports the assumption of an analogue synthesis pathway starting from homomethionine as published in the work by Rapp et al. [42]. As can be seen from the results presented in Table 3, wines from Zelina had significantly the highest amount of 4-methylthio-1-butanol while wines from Plešivica stood out with significantly the highest concentrations of 3-methylthio-1-propanol. Among volatile components lactones, mainly *γ*-lactones and whiskey lactones can influence wine aroma by adding “fruity”, “coconut-like” and “peach-like” notes. Lactones mostly arise from cyclisation of the corresponding *γ*-hydroxycarboxylic acids, an unstable molecules that can be formed by glutamic acid deamination and decarboxylation process [22,39,43]. Lactones may also come from grapes, as is the case in Riesling, where they contribute to the varietal aroma [24]. The concentration of lactones in thirteen samples of sparkling wine were analysed by Kosmerl and Cegnar [44], with values between 15.0 and 57.5 µg L^−1^, and *γ*-nonalactone and *γ*-decalactone with levels below 4.7 µg L^−1^. In contrast, in Croatian sparkling wines values were much higher, ranging between 7.58 and 25.44 µg L^−1^ for *γ*-nonalactone and 1.93 and 57.19 µg L^−1^ for *γ*–decalactone while *γ*-octalactone concentrations were lower, between 1.06 and 1.80 µg L^−1^. Comparing average lactones concentrations between vine-growing regions significantly higher values were determined in sparkling wines from Plešivica primarily due to teh presence of *γ*-butyrolactone. Among others, significantly, the highest concentrations of *γ*-decalactone and *γ*-undecalactone were detected in sparkling wines from Krašić.

### 2.3. Multivariate Analysis

Discriminant analysis using forward stepwise model for all volatile compounds showed that 21 volatile compounds were selected and ranked based on their discrimination efficiency of three vine-growing regions (Table 4), while other compounds were not included by the model as the threshold to enter was set to 0.05.

Three vine-growing regions can be clearly separated, and that Fisher distances between all of them are significant (Table 5).

**Figure 1:**
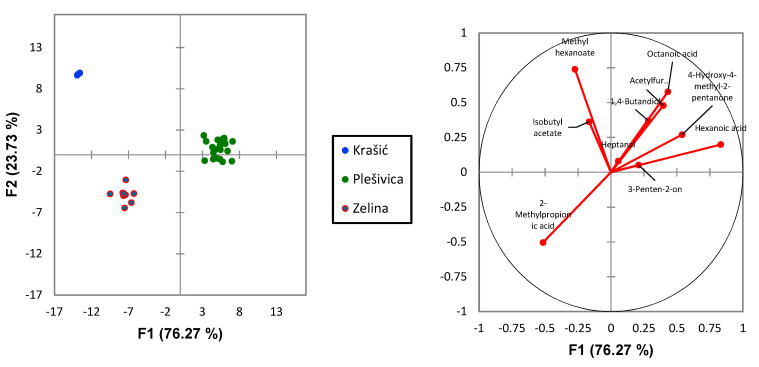
Discriminant analysis of 30 sparkling wines (**left**) based on the concentrations of ten volatile compounds (**right**)) with highest rank after forward stepwise model applied on total number of volatile compounds detected. The vector diagram indicates the direction and intensity of the effect of nine variables on the distribution of wine samples in the space defined by two discrimination factors (F1 and F2).

Scatter plot presented in Figure 1 obtained on the basis of the discriminant analysis showed the distribution of the sparkling wines in the space defined with two discrimination factors. Based on the vector diagram of ten highest ranked volatile compounds selected using forward stepwise model in discriminant analysis we can conclude that due to the position of the plot, samples from Plešivica vine-growing region are specific for high content of hexanoic acid, octanoic acid, 4-hydroxy−4-methyl-2-pentanon, 1,4-butandiol, and acetylfurane, samples from Zelina are specific for a higher content of 2-methylpropionic acid, while Krašić samples have higher level of methyl hexanoate and isobutyl acetate. After the series of discriminant analyses were performed starting with two variables with the highest rank based on stepwise discriminant analysis, followed by the introduction of one new variable in each new analysis, we have discovered that using first two variables (methyl hexanoate and hexanoic acid) 100% correct classification was achieved for Krašić and Zelina while 95% correct classification was achieved for Plešivica samples. After one additional variable (4-hydroxy-4-methyl-2-pentanone) was included, all samples were classified within the corresponding vine-growing region. Significant Fisher distances were detected (Table 5) among all three groups using these three variables (methyl hexanoate, hexanoic acid, and 4-hydroxy-4-methyl-2-pentanone).

In the past years’ classification of musts and/or wines by multivariate analysis were carried on on the basis of their geographical origin or viticultural region [14], based on their chemical attributes [74] and aroma profile [66]. In the work by Arozarena et al. [76] discriminant selection process showed that correct classification by grape cultivar was achieved in the 94% of the training wines and 85% of the test wines. These percentages were very similar when the separation model was used to test the differences between regions, achieving 89% in training sample set and 92% in test wines samples. Similar results were achieved by Marais et al. [77] where stepwise discriminant analysis was applied for the separation based on the aroma compounds data of dry white table wine. The highest discriminatory value components were isoamyl and hexyl acetate and isobutanol, in the Colombar wines while in the Chenin blanc wines they were 2-phenyl ethanol and hexanol.

### 2.4. Odour Active Values (OAV) and Relative Odour Contribution (ROC)

To evaluate the influence of individual volatile compounds on overall aroma of sparkling wines from three vine-growing regions, OAV values and ROC indexes were calculated and presented in Table 6. From the total of 174 compounds only 26 exceeded the treshold values (OAV > 1). Between them, the most abundand were esters with seven individual compounds and aldehydes with five compounds. The highest OAV was noted in Krašić samples where isoamly lactate OAV value was notably higher compared to other two vine-growing regions. Among others hexanal and β-damascenone stood up in Zelina samples while in the sparkling wines from Plešivica vine-growing region hexanoic acid OAV was the highest. ROC index pointed out pronounced influence of volatile compounds belonging to esters and aldehydes in all sparkling wine samples.

## 3. Materials and Methods

### 3.1. Samples

Commercial Croatian sparkling wines samples (*n* = 30), were obtained from the wineries located in Plešivica, Zelina and Krašić vine-growing regions, all within Zagreb County. In Plešivica vine-growing region the dominant grape varieties were Chardonnay, Pinot noir and Portugizer, in Zelina beside Pinot noir and Chardonnay the presence of Kraljevina (autohotnous Croatian variety) was notable while in Krašić for all the sparkling wines Manzoni bianco grape variety was used. Grapes used for the sparkling wines production were manually harvested while second fermentation was conducted in bottles for the period of 9 months. The first step, before chemical analysis, was to degas sparkling wines by use of Sonorex Ultrasonic bath (Bandelin ElectronicGmbH & Co. KG, Berlin, Germany). To eliminate carbon dioxide, approximately 50 mL of sparkling wines sample was put in a centrifuge tube and degassed for around 2 min.

### 3.2. Volatile Compounds Determination

Wine sample volatile compounds analysis was performed according to the described method [78]. Isolation of analytes was performed by solid phase extraction on LiChrolut EN cartridges (200 mg/3 mL, Merck, Darmstadt, Germany). In the column which was previously conditioned by successive washing with 3 mL dichloromethane (UHPLC gradient grade J.T.Baker, Deventar, The Netherland), methanol (UHPLC gradient grade J.T.Baker, Deventar, The Netherland) and a 13% aqueous ethanol (LiChrosolv, Merck, Darmstadt, Germany) solution 50 mL of sample was loaded. After the passage of the sample through column, residual sugars and other polar compounds were washed out by 3 mL of water. The column was dried by passing the air. The eluation of analytes was done by 1 mL of dichloromethane. As a quality control, 50 mL of water was injected to the SPE column instead of the sample. Quantitative and qualitative analysis was performed on an Agilent 6890 system coupled with 5973N mass spectrometer with the column ZB-WAX (60 m × 0.32 mm i.d., with 0.5 µm film thickness, Phenomenex, Torrance, USA). The temperature program was as follow 40 °C for 15 min, from 40 to 250 °C with increments of 2 °C per minute and 250 °C for 15 min. Transfer line was set to 250 °C, the flow rate of helium was 1 mL min^−1^. The MS was operated in electron ionization (EI) mode at 70 eV with a total ion current (TIC) monitoring. Identification was done by comparing retention times and mass spectra with those of standards. List of used standards, linear retention indices and other parameters for identification and quantification is presented in Table 7. Quantification was done by calibration curves. The curves (based on quantification ions) were constructed with software Enhanced ChemStation (Agilent Technologies, Santa Clara, CA, USA, SAD). For all available standards (172) six different concentrations were prepared. For two compounds (Terpendiol I and II) semi-quantitative analysis was performed. Their concentrations were expressed in equivalents of similar compounds with assumption that a response factor was equal to one.

### 3.3. Determination of Organic Acids

Analysis of individual acids (tartaric, malic and citric acid) were done by HPLC system Agilent Series 1100 equipped with Diode Array Detector (Agilent, Palo Alto, CA, USA). In brief, the determination was performed isocratically with a flow rate set to 0.6 mL min^−1^ with 0.065 % phosphoric acid (p.a. Merck, Darmstadt, Germany) as a mobile phase. Column Aminex HPX-87H 300 × 7.8 mm i.d (Bio-Rad Laboratories, Hercules, CA, USA) was heated at 65 °C, while the detector was set to 210 nm.

### 3.4. Determination of Odour Activity Values and Relative Odour Contributions

Each chemical substance can have specific influence on the wine aroma. It can be presented by the odour activity value (OAV) and relative odour contributions (ROC). So they can be used as a markers in determining the role of a specific compound in the sample aroma composition. OAV is calculated as the quotients of their concentration (c) and the corresponding odour detection threshold (t) reported in the literature [79]. Volatile aroma substances with an OAV ≥ 1 can have direct impact on aroma and they are usually marked as one of the most significant volatile substances or the most active odours [80]. The volatiles with an OAV < 1 can also positively influence the wine aroma complexity and aromatic intensity of other compounds through synergistic effects. The ROC of each aroma compound is calculated as the ratio of the OAV of the respective compound to the total OAV of each wine [81].

### 3.5. Statistical Analysis

The analysis of variance was used for the statistical assessment of the data and Duncan’s multiple range test was used to determine significant differences (*p* < 0.05) among means. Multivariate analysis was carried out with XLSTAT software v.2020.3.1. (Addinsoft, New York, NY, USA). The forward stepwise model was used to select and rank the variables based on contribution to the discrimination of the groups. The selection process starts by using the variable with the largest contribution to the model and then the following variable is added with an enter probability greater than the threshold value. When the third and all the following variables are being added, model then evaluate the impact of removing each previously present variable in contrast to the removal threshold. To test the minimal number of dependent variables required to achieve the 100% correct classification using cross-validation of the samples within the belonging group, i.e., vine-growing region, a series of discriminant analyses were performed using all of the samples, starting with two variables with the highest rank based on stepwise discriminant analyses, and in each new analysis, one new variable was added as a differentiating factor among cultivars. This determined the cumulative efficiency of the parameters applied in the correct classification of wine samples in the corresponding vine-growing region.

## 4. Conclusions

Even though in the current work little was known about the enological steps used in the production of the wines studied, differences were clearly demonstrated and wines classified according to the vine-growing regions, indicating that future studies using greater control over enological factors are likely to demonstrate an even stronger role of the site in the sparkling wine composition. As can be seen from the results, in all sparkling wines, esters had an important role, among them especially the once with the OAV > 1 as ethyl butanoate, hexanoate, octanoate, ethyl-2-methylbutanoate, ethyl-3-methylbutanoate, isoamyl acetate, and isoamyl lactate. The presence of diethyl succinate as well as diethyl glutarate, compounds whose presence can be used as an ageing marker was detected. Another compound that could be used as discriminate marker is TDN whose concentrations were notably higher in sparkling wines from Zelina vine-growing region. Such data could lead to a better understanding of what defines sparkling wines of a specific vine-growing region. However, this work provides a basis for the future research variations of volatile aroma compounds within Croatian sparkling wines from Zagreb County and for the development of models that better explain these variations due to the geographic origin that is associated with similar climatic conditions or soil.

## Figures and Tables

**Table 1:** Organic acid composition (g L^−1^) of sparkling wines from different vine-growing regions.

Parameters	Vine Growing Regions								
	Plešivica (*n* = 19)			Zelina (*n* = 8)			Krašić (*n* = 3)		
	MIN	MAX	Mean ± SD	MIN	MAX	Mean ± SD	MIN	MAX	Mean ± SD
Tartaric acid	1.28	3.77	2.35 ± 0.70	1.72	2.91	2.23 ± 0.40	1.61	2.50	2.06 ± 0.62
Malic acid	0.12	1.61	0.81 ± 0.43	0.43	2.02	1.20 ± 0.55	0.99	1.62	1.31 ± 0.44
Citric acid	0.04	0.51	0.18 ± 0.12	0.06	0.59	0.26 ± 0.15	0.06	0.08	0.07 ± 0.01
Succinic acid	0.01	0.50	0.13 ± 0.14	0.01	0.28	0.11 ± 0.08	0.03	0.07	0.05 ± 0.02
Lactic acid	0.01	0.27	0.10 ± 0.09	0.07	0.36	0.16 ± 0.09	0.10	0.13	0.12 ± 0.09
pH value	2.89	3.56	3.16 ± 0.17	3.05	3.39	3.16 ± 0.10	3.20	3.45	3.33 ± 0.10

MIN-minimum value, MAX-maximum value, SD-standard deviation.

**Table 2:** Average volatile compound concentrations (µg L^−1^) of sparkling wines produced in different vine-growing regions.

Parameters	Vine Growing Regions								
	Plešivica (*n* = 19)			Zelina (*n* = 8)			Krašić (*n* = 3)		
	MIN	MAX	Mean ± SD	MIN	MAX	Mean ± SD	MIN	MAX	Mean ± SD
∑ Aldehydes	356.26	1902	842 ± 62 ^ab^	281.36	1161	546.77 ± 88.03 ^c^	978.02	1100	1039 ± 172 ^a^
∑ Higher alcohols	38338	59964	49531 ± 5846 ^b^	44334	97754	75423 ± 20673 ^a^	42223	43706	42965 ± 1048 ^b^
∑ Volatile phenols	84.0	448.1	181.6 ± 89.0 ^b^	17.1	257.1	84.5 ± 76.1 ^c^	304.5	458.0	381.3 ± 108.5 ^a^
∑ Terpenes	188.6	1270	475.3 ± 242.0 ^b^	481.8	1044	760.0 ± 219.8 ^a^	609.9	800.6	705.3 ± 134.8 ^ab^
∑ C13-norisoprenoides	0.3	13.1	3.9 ± 4.7 ^b^	4.1	13.2	8.1 ± 2.7 ^a^	0.1	1.5	0.84 ± 1.0 ^c^
∑ Lactones	16.97	740.33	158.96 ± 218.0 ^a^	15.83	472.28	133.34 ± 179.8 ^b^	22.09	180.32	101.20 ± 111.8 ^b^
∑ Esters	14990	30215	21658 ± 4660 ^b^	15604	85050	48045 ± 29098 ^a^	16456	23977	20217.3 ± 5318 ^b^
∑ Fatty acids	6815	25035	15244 ± 5327 ^a^	183.8	8970	2137 ± 3324 ^b^	6915	10363	8639 ± 2438 ^ab^
∑ Other alcohols	419.6	2381.8	947.1 ± 478.1 ^a^	140.2	815.0	302.4 ± 241.9 ^b^	637.0	769.0	703.0 ± 93.3 ^ab^
∑ Sulfur compounds	27.64	195.5	103.8 ± 40.3 ^b^	96.4	932.9	574.9 ± 302.5 ^a^	30.2	65.5	47.9 ± 24.9 ^b^
∑ Other compounds	102.69	3025	553.4 ± 750.0 ^a^	82.3	291.6	162.6 ± 79.9 ^a^	269.3	332.3	300.9 ± 44.5 ^a^

MIN-minimum value, MAX-maximum value, SD-standard deviation; Means mean ± S.D. are calculated only from samples in which analytes were quantified; Means with different superscript letters in the same row differ significantly (*p* ≤ 0.05).

**Table 3:** Individual volatile compound concentrations (µg L^−1^) of sparkling wines produced in different vine growing regions.

Parameters	ODT (µg L^−1^)	Odour Descriptor	OAV	Vine-Growing Regions								
				Plešivica			Zelina			Krašić		
				MIN	MAX	Mean ± SD	MIN	MAX	Mean ± SD	MIN	MAX	Mean ± SD
Aldehydes												
2,4-Decadienal	270 [45]	floral [46]		0.03	8.80	2.19 ^ab^ ± 1.89	n.d.	3.67	1.47 ^b^ ± 1.23	2.72	3.28	3.00 ^a^ ± 0.23
2,4-Heptadienal *(E,E)*				0.05	23.61	6.30 ^a^ ± 5.70	n.d.	11.11	4.0 ^a^ ± 4.94	8.95	9.90	9.43 ^a^ ± 0.39
2,4-Heptadienal *(Z,Z)*				0.07	9.35	3.75 ^a^ ± 2.52	n.d.	12.06	11.30 ^a^ ± 19.69	6.31	16.98	11.65 ^a^ ± 4.36
2,4-Nonadienal	0.09 [47]	cucumber [46]	>1	0.12	4.73	1.70 ^a^ ± 1.26	n.d.	5.74	0.70 ^a^ ± 1.78	0.22	3.56	1.89 ^a^ ± 1.36
2-Heptenal	4.6 [48]	green [46]	>1	n.d.	383.08	215.13 ^a^ ± 92.35	n.d.	153.46	17.1 ^b^ ± 48.23	147.88	156.95	152.42 ^a^ ± 3.70
5-Hydroxymethylfurfural	100,000 [48]	almond [49]		1.30	789.45	67.40 ^b^ ± 166.44	0.26	35.44	7.98 ^b^ ± 12.06	57.87	453.56	255.72 ^a^ ± 161.54
5-Methyl-furfural	16,000 [48]	bitter, almond, spice [49]		2.38	35.45	10.28 ^a^ ± 7.29	2.75	13.96	7.57 ^b^ ± 3.40	7.21	15.69	11.45 ^a^ ± 3.46
Benzeneacetaldehyde	4 [50]			81.5	246.35	142.60 ^a^ ± 49.27	n.d.	318.1	70.92 ^a^ ± 109.14	110.87	202.25	156.56 ^a^ ± 37.31
Benzaldehyde	350 [51]	bitter, almond [52]		8.71	78.56	27.23 ^a^ ± 17.87	2.91	50.96	20.38 ^a^ ± 14.50	30.9	35.25	33.08 ^a^ ± 1.78
Decanal	0.1–2 [53]		>1	n.d.	138.25	25.71 ^a^ ± 42.02	0.89	117.58	55.23 ^a^ ± 34.39	0.25	51.77	26.01 ^a^ ± 21.03
Furfural	770 [54]	burn, almond, yeast [55]		5.24	423.08	125.89 ^a^ ± 108.98	n.d.	279.63	164.93 ^a^ ± 87.47	154.58	291.47	223.03 ^a^ ± 55.89
Heptanal	3 [53]			n.d.	3.05	0.84 ^a^ ± 0.75	n.d.	2.79	0.75 ^a^ ± 0.98	0.81	0.93	0.87 ^a^ ± 0.05
Hexanal	5 [56]	herbaceous [51]	>1	90.13	428.31	203.69 ^b^ ± 90.81	221.77	433.73	315.25 ^a^ ± 64.33	100.29	173.01	136.65 ^b^ ± 29.69
Nonanal	1 [50]		>1	n.d.	19.45	10.04 ^a^ ± 7.29	n.d.	17.38	3.65 ^b^ ± 2.77	15.12	19.82	17.47 ^a^ ± 6.77
Higher alcohols												
1-Butanol	150,000 [57]	medicinal [51]		43.07	186.43	112.11 ^a^ ± 42.20	28.48	78.64	57.75 ^b^ ± 14.98	93.06	109.75	101.41 ^ab^ ± 6.81
1-Decanol	5000 [58]	pear, waxy, violet [58]		0.44	1.75	0.69 ^b^ ± 0.32	0.46	6.17	3.83 ^a^ ± 2.08	0.42	0.67	0.55 ^b^ ± 0.10
1-Heptanol	425 [50]	oily [46]		2.72	64.47	19.21 ^a^ ± 18.05	0.98	13.19	4.37 ^b^ ± 4.64	26.91	31.31	29.11 ^a^ ± 1.80
1-Hexanol	2500 [55]	grass just cut [51]	>1	677	3707	1874 ^b^ ± 788	471	3260	1612 ^b^ ± 848	2719	3177	2948 ^a^ ± 186
1-Methoxy-2-propanol				8.34	82.26	19.82 ^a^ ± 15.70	n.d.	17.24	4.78 ^b^ ± 6.42	13.54	25.96	19.75 ^ab^ ± 5.07
1-Octadecanol				0.41	0.81	0.59 ^a^ ± 0.10	0.58	3.17	2.24 ^a^ ± 1.09	0.7	15.94	8.32 ^a^ ± 6.22
1-Octanol	110–130 [53]	chemical [51]		3.20	8.37	5.29 ^b^ ± 1.47	5.16	8.65	6.74 ^a^ ± 1.23	5.43	5.92	5.68 ^ab^ ± 0.20
1-Pentanol	64,000 [39]	bitter, almond, balsamic [39]		9.34	31.92	15.82 ^a^ ± 5.09	0.30	24.3	6.90 ^b^ ± 9.30	15.95	18.07	17.01 ^a^ ± 0.87
2-Heptanol	70 [59]	fruity, herbaceous [46]		2.85	24.47	7.66 ^a^ ± 4.56	0.40	10.07	6.66 ^a^ ± 4.89	6.51	6.74	6.63 ^a^ ± 0.09
*trans*-2-Hexene-1-ol	100 [59]	herbaceous, green [46]		0.59	10.31	2.96 ^b^ ± 2.86	1.86	15.25	8.53 ^a^ ± 5.49	1.14	2.15	1.65 ^b^ ± 0.41
*cis*-2-Hexene-1-ol		green [46]		9.21	29.58	15.74 ^b^ ± 5.94	4.18	17.95	11.19 ^b^ ± 4.47	20.8	32.56	26.68 ^a^ ± 4.80
2-Methyl-1-butanol	30,000 [60]	whiskey, burnt, nail polish [61]		9245	13,171	10835 ^b^ ± 1180	6898.	22720	17390 ^a^ ± 6395	8031	9147	8589 ^b^ ± 455
2-Pentadecanol				n.d.	4.50	0.80 ^b^ ± 1.31	n.d.	3.52	1.90 ^a^ ± 1.39	-	-	n.d.
2-Pentene-1-ol				1.84	18.92	4.75 ^b^ ± 3.97	4.63	28.41	18.61 ^a^ ± 9.04	3.38	3.78	3.58 ^b^ ± 0.16
*cis*-3-Hexene-1-ol	400 [51]	grass, green [51]		29.46	678.41	102.16 ^a^ ± 132.05	52.76	343.47	146.75 ^a^ ± 88.32	77.12	240.48	158.80 ^a^ ± 66.69
*trans*-3-Hexene-1-ol	1000 [51]	grass, resinous, cream [51]		23.69	212.65	81.96 ^a^ ± 56.82	1.24	116.61	53.84 ^a^ ± 30.47	48.97	53.12	51.05 ^a^ ± 1.69
3-Octanol				n.d.	2.45	1.26 ^a^ ± 0.98	n.d.	4.97	2.22 ^a^ ± 1.64	0.10	2.32	1.21 ^a^ ± 0.91
Isoamyl alcohol	30,000 [60]	alcohol, nail polish [58]	>1	16317	29862	23888 ^b^ ± 2628	25039	53991	39580 ^a^ ± 10019	20291	20724	20507 ^b^ ± 176
Isobutanol	40,000 [39]	alcohol, nail polish [58]		1467	5273	2686 ^a^ ± 1013	n.d.	3042	937 ^b^ ± 1330	2403	2467	2435 ^ab^ ± 26
Phenylethyl alcohol	14,000 [62]	floral, rose, honey [58]	>1	2950	12347	9734 ^ab^ ± 2077	5315	26107	15759 ^a^ ± 7467	7349	8731	8040 ^b^ ± 564
Volatile phenols												
4-Ethylguaiacol	33 [39]	toasted bread, smoky, clove [51]		2.37	23.44	7.38 ^b^ ± 5.36	5.35	56.60	26.68 ^a^ ± 18.97	7.93	14.49	11.21 ^ab^ ± 2.68
4-Ethylphenol	35 [63]	phenol, stable [51]		n.d.	146.86	13.35 ^a^ ± 37.64	-	-	n.d.	0.11	88.68	44.40 ^a^ ± 36.16
4-Vinylguaiacol	40 [63]	clove, curry [51]	>1	40.57	207.87	114.75 ^b^ ± 40.41	0.06	166.26	28.44 ^c^ ± 57.37	135.81	377.52	256.67 ^a^ ± 98.68
4-Vinylphenol	180 [62]	phenolic, medicinal [51]		n.d.	0.42	0.08 ^b^ ± 0.09	n.d.	0.19	0.10 ^b^ ± 0.11	n.d.	1.35	0.68 ^a^ ± 0.55
Eugenol	6 [54]	cinnamon, clove [51]		0.04	8.13	1.79 ^a^ ± 1.91	0.03	0.32	0.18 ^a^ ± 0.14	0.09	3.80	1.95 ^a^ ± 1.51
Guaiacol	9.5 [55]	smoky, hospital [55]		0.07	5.76	0.97 ^a^ ± 1.32	n.d.	0.32	0.20 ^a^ ± 0.31	0.13	1.03	0.58 ^a^ ± 0.37
Homovanillyl alcohol				-	-	n.d.	n.d.	4.18	1.69 ± 1.34	-	-	n.d.
Vanillin	200 [60]	vanilla [51]		1.06	278.59	32.77 ^a^ ± 57.53	3.38	71.33	16.98 ^a^ ± 21.89	60.34	63.09	61.72 ^a^ ± 1.12
Tyrosol	33 [39]	toasted bread, smoky, clove [39]		1.45	15.39	7.50 ^a^ ± 4.30	1.35	30.38	11.07 ^a^ ± 8.82	3.73	4.49	4.11 ^a^ ± 0.31
Terpenes												
1,8-Terpin				0.22	9.93	2.74 ^a^ ± 3.01	0.65	3.88	1.68 ^a^ ± 15.05	0.54	2.52	1.53 ^a^ ± 0.81
6,7-Dihydro-7-hydroxylinalool				0.11	80.02	24.31 ^a^ ± 27.38	10.33	56.2	39.41 ^a^ ± 250.41	0.43	55.03	27.73 ^a^ ± 22.29
8-Hidroxylinalool				0.91	74.75	23.71 ^a^ ± 19.55	1.54	20.52	11.85 ^a^ ± 11.01	7.31	13.44	10.38 ^a^ ± 2.50
*α*-Terpineol	330 [64]	lilac, floral, sweet [51]		6.52	51.93	21.04 ^a^ ± 12.52	9.32	44.34	26.05 ^a^ ± 165.20	24.41	52.36	38.39 ^a^ ± 11.41
*β*-Farnesen	87 [65]			0.07	8.50	2.15 ^a^ ± 2.78	n.d.	0.51	0.27 ^a^ ± 0.16	0.44	0.59	0.52 ^ab^ ± 0.06
*β*-Ocimene				0.17	2.00	0.87 ^b^ ± 0.63	n.d.	0.76	0.23 ^b^ ± 0.46	1.17	1.68	1.43 ^a^ ± 0.21
*cis*-Linalool oxide, furan	6000 [55]	flower [55]		0.10	20.13	10.85 ^a^ ± 6.87	2.28	23.15	12.04 ^a^ ± 38.62	17.69	30.44	24.07 ^a^ ± 5.21
Citronelol	40 [64]	rose [66]		1.86	19.15	4.59 ^a^ ± 3.81	n.d.	5.48	1.46 ^a^ ± 2.13	6.4	13.36	9.88 ^a^ ± 2.84
*δ*-Carene				n.d.	1.73	0.58 ^a^ ± 0.49	n.d.	3.54	2.51 ^a^ ± 1.70	0.35	2.53	1.44 ^a^ ± 0.89
Dihydroactinidiolide				n.d.	22.57	7.69 ^a^ ± 5.79	n.d.	6.91	2.87 ^a^ ± 2.22	n.d.	n.d.	n.d.
Farnesol	20 [67]	floral, clove [46]		0.77	14.25	5.73 ^a^ ± 4.65	0.14	9.22	3.06 ^a^ ± 2.68	7.98	8.08	8.03 ^a^ ± 0.04
*γ*-Terpinene				n.d.	90.92	7.79 ^b^ ± 22.99	0.40	240.91	131.94 ^a^ ± 88.91	0.32	0.41	0.37 ^b^ ± 0.04
Geraniol	20 [62]	citrus, citric fruit [51]	>1	0.35	16.52	2.21 ^b^ ± 3.38	0.58	152.65	88.33 ^a^ ± 52.92	1.57	14.28	7.93 ^b^ ± 5.19
Geranyl acetate	9 [68]	flowery [68]		0.11	17.91	3.65 ^a^ ± 3.84	n.d.	11.19	5.97 ^a^ ± 18.23	2.65	6.58	4.62 ^a^ ± 1.60
Hotrienol	110 [52]	fresh, floral, fruity [52]	>1	7.69	121.11	31.94 ^a^ ± 28.55	1.68	142.97	29.30 ^a^ ± 607.88	128.35	268.59	198.47 ^a^ ± 57.25
Linalool	25 [62]	citrus, floral, sweet [51]	>1	0.69	103.38	11.31 ^b^ ± 23.72	1.38	84.8	45.03 ^a^ ± 30.83	1.3	144.91	73.11 ^a^ ± 58.63
Linalool oxide, pyran	3000 [55]	flower [51]		2.18	13.23	7.17 ^a^ ± 3.37	0.62	19.77	6.54 ^a^ ± 133.08	22.65	26.44	24.55 ^a^ ± 1.55
Neric acid				1.87	149.88	26.84 ^a^ ± 40.12	1.93	14.99	7.42 ^a^ ± 4.88	32.44	40.05	36.25 ^a^ ± 3.11
Nerol	300 [64]	rose, fruity, floral [51]		0.12	6.13	0.91 ^b^ ± 1.23	0.17	60.41	33.19 ^a^ ± 20.17	0.77	3.09	1.93 ^b^ ± 0.95
Nerolidol	250 [50]	rose, apple, green, waxy, woody [46]		0.11	3.22	0.57 ^a^ ± 0.64	0.10	3.58	1.28 ^a^ ± 1.44	0.01	0.67	0.34 ^a^ ± 0.27
Pseudoionon				n.d.	0.14	0.06 ^b^ ± 0.03	n.d.	1.49	0.09 ^b^ ± 0.49	0.08	1.53	0.81 ^a^ ± 0.59
Terpendiol I				n.d.	1.89	0.74 ^a^ ± 0.53	n.d.	0.98	0.87 ^a^ ± 0.80	0.8	1.18	0.99 ^a^ ± 0.16
Terpendiol II				2.40	40.50	11.30 ^c^ ± 9.32	0.81	48.83	31.69 ^b^ ± 39.30	53.43	114.59	84.0 ^a^ ± 24.97
Terpinene-4-ol				0.03	3.97	2.25 ^b^ ± 0.85	1.79	91.4	36.23 ^a^ ± 29.88	1.73	3.5	2.62 ^b^ ± 0.72
Tetrahydrolinalool				0.1	1015	183.23 ^a^ ± 226.07	68.33	436.64	198.41 ^a^ ± 125.28	1.26	53.42	27.34 ^b^ ± 21.29
*trans*-Linalool oxide, furan	6000 [55]	flower [55]		0.25	105.39	13.87 ^a^ ± 21.89	1.15	50.99	20.81 ^a^ ± 54.31	18.23	49.27	33.75 ^a^ ± 12.67
*trans*-Rose oxide	80–160 [69]	floral, green [69]		0.11	0.39	0.20 ^a^ ± 0.12	0.10	0.24	0.24 ^a^ ± 0.45	0.24	0.48	0.36 ^a^ ± 0.10
Linalyl formate				n.d.	4.69	0.81 ^b^ ± 1.04	0.41	5.99	2.61 ^a^ ± 2.06	0.27	0.64	0.46 ^b^ ± 0.15
Ethyl linalyl acetal				0.64	5.74	2.62 ^b^ ± 1.41	1.01	28.94	9.90 ^a^ ± 8.92	1.92	2.45	2.19 ^b^ ± 0.22
2,6-Dimethyl-3,7-octadiene-2,6-diol				0.07	12.17	3.77 ^b^ ± 3.29	0.20	14.03	5.80 ^b^ ± 25.31	20.2	30.78	25.49 ^a^ ± 4.32
2,6-Dimethyl-7-octene-2,6-diol				25.16	80.60	46.12 ^a^ ± 15.09	8.06	40.19	16.79 ^a^ ± 14.38	48.81	54.76	51.79 ^a^ ± 2.43
Menthol				0.93	57.06	9.97 ^a^ ± 17.41	n.d.	1.61	0.55 ^a^ ± 1.67	2.21	3.71	2.96 ^a^ ± 0.61
Ocimenol				n.d.	2.47	0.95 ^a^ ± 0.65	n.d.	5.59	3.96 ^a^ ± 7.46	0.59	1.42	1.01 ^a^ ± 0.34
C13- norisoprenoids												
*α*-Ionol				0.01	0.78	0.24 ^a^ ± 0.21	n.d.	0.09	0.07 ^b^ ± 0.13	0.05	0.18	0.12 ^ab^ ± 0.05
*α*-Ionon	10.5 [70]	floral [70]		n.d.	0.05	0.03 ^b^ ± 0.01	0.18	0.56	0.37 ^a^ ± 0.18	0.02	0.06	0.04 ^b^ ± 0.02
*β*-Damascenone	0.05 [60]	sweet, fruity, floral, honey [62]	>1	0.05	9.95	1.91 ^a^ ± 2.95	0.03	6.54	3.68 ^a^ ± 2.14	n.d.	0.28	0.14 ^a^ ± 0.11
TDN	2 [71]	petrol, kerosene [55]	>1	0.35	8.86	2.55 ^a^ ± 2.26	0.1	7.45	3.89 ^a^ ± 2.72	0.1	1.1	0.55 ^a^ ± 0.45
Lactones												
*cis*-Whiskey lactone	67 [39]	nutty, coconut [61]		0.46	5.31	1.59 ^a^ ± 1.19	2.54	4.64	2.13 ^a^ ± 1.25	0.74	0.81	0.78 ^a^ ± 0.03
*δ*-Nonalactone				5.51	13.07	8.71 ^a^ ± 2.04	n.d.	8.73	2.86 ^b^ ± 2.92	7.59	8.11	7.85 ^a^ ± 0.21
*γ*-Butyrolactone	10,000 [58]	coconut, caramel [51]		n.d.	695.54	131.27 ^a^ ± 208.88	n.d.	439.29	85.94 ^a^ ± 227.18	-	-	n.d.
*γ*-Decalactone	1000 [58]	peach, fruity [51]		0.55	7.09	1.93 ^b^ ± 1.45	n.d.	7.34	5.12 ^b^ ± 2.37	1.93	112.45	57.19 ^a^ ± 45.12
*γ*-Hexalactone	1600 [72]	sweet, cake, peach [51]		0.19	28.81	5.80 ^a^ ± 5.71	n.d.	6.22	3.33 ^a^ ± 2.37	n.d.	13.82	6.91 ^a^ ± 5.64
*γ*-Nonalacton	25 [48]	coconut, peach [51]	>1	2.67	23.85	7.58 ^b^ ± 5.27	5.06	49.11	25.44 ^a^ ± 17.36	7.95	14.66	11.31 ^ab^ ± 2.74
*γ*-Octalacton	7 [48]			n.d.	8.48	1.06 ^a^ ± 1.78	n.d.	2.45	1.77 ^a^ ± 0.80	1.74	1.85	1.80 ^a^ ± 0.04
*γ*-Undecalactone	60 [48]	apricot, peach [46]		0.15	1.84	0.54 ^b^ ± 0.44	n.d.	4.66	2.21 ^b^ ± 1.68	2.01	28.55	15.28 ^a^ ± 10.84
*trans*-Whiskey lactone	790 [39]	nutty, coconut [61]		0.10	26.93	2.21 ^a^ ± 5.58	2.37	2.65	1.53 ^a^ ± 0.86	0.07	0.14	0.11 ^a^ ± 0.03
Esters												
2-Phenylethyl acetate	250 [73]	rose, honey, tobacco [58]		7.04	115.22	23.40 ^a^ ± 22.55	0.16	41.73	24.05 ^a^ ± 14.97	10.14	47.31	28.73 ^a^ ± 15.17
Diethyl glutarate				n.d.	28.02	15.39 ^a^ ± 8.21	5.32	35.62	19.93 ^a^ ± 10.19	5.73	18.29	12.01 ^a^ ± 5.13
Diethyl hydroxysuccinate				n.d.	5.42	1.26 ^b^ ± 1.96	0.89	69609	32046 ^a^ ± 27325.14	n.d.	11.81	5.91 ^b^ ± 4.82
Diethyl malonate		sweet, fruity, apple [46]		0.93	13.03	7.08 ^b^ ± 2.94	4.09	10.95	8.55 ^b^ ± 2.34	13.23	46.31	29.77 ^a^ ± 13.50
Diethyl oxalate				n.d.	1.31	0.34 ^a^ ± 0.35	n.d.	0.96	0.48 ^a^ ± 0.30	-	-	n.d.
Diethyl succinate	200,000 [39]	overripe, aged [55]		1600	10272	5401 ^a^ ± 2141	3141	11498	7430 ^a^ ± 2545	2700	5134	3917 ^b^ ± 993
Dimethyl malate				0.10	10.03	3.82 ^b^ ± 3.08	-	-	n.d.	n.d.	15.61	7.81 ^a^ ± 6.37
Ethyl benzeneacetate				6.76	77.85	34.36 ^a^ ± 23.91	0.97	71.60	11.80 ^b^ ± 25.34	16.4	40.00	28.20 ^ab^ ± 9.63
Ethyl butanoate	20 [54]	pineapple, apple, peach [58]	>1	117.13	404.93	270.94 ^b^ ± 82.85	204.06	580.88	417.08 ^a^ ± 119.30	352.95	372.23	362.59 ^ab^ ± 7.87
Ethyl decanoate	200 [63]	floral, grape, fruty [61]		0.10	150.72	9.32 ^b^ ± 32.79	0.16	239.31	136.02 ^a^ ± 85.11	0.18	0.20	0.19 ^b^ ± 0.01
Ethyl furoate	16,000 [54]			6.85	81.43	43.46 ^a^ ± 18.89	2.51	111.91	21.80 ^a^ ± 34.42	32.66	46.97	39.82 ^a^ ± 5.84
Ethyl hexadecanoate	>2000 [74]			0.17	2.50	0.94 ^b^ ± 0.73	0.30	16.27	6.54 ^a^ ± 6.04	0.24	1.46	0.85 ^b^ ± 0.50
Ethyl hexanoate	14 [62]	fruity, green apple, banana [61]	>1	111.89	578.37	334.98 ^b^ ± 129.46	181.96	803.90	520.55 ^a^ ± 190.77	423.93	447.10	435.52 ^ab^ ± 9.46
Ethyl hydrogensuccinate				4096	9546	6671 ^a^ ± 1754	0.35	7160	2346.13 ^b^ ± 314.10	2692	6870	4781 ^ab^ ± 1705
Ethyl lactate	154,000 [54]	butter [58]		2131	14871	6664 ^a^ ± 3755	2645	7325	4025 ^b^ ± 2275	8315	9128	8721 ^a^ ± 331
Ethyl linoleate	450 [65]			0.19	1.91	0.68 ^b^ ± 0.41	0.54	3.00	2.00 ^a^ ± 0.79	0.44	1.01	0.73 ^b^ ± 0.23
Ethyl octanoate	580 [63]	sweet, floral, fruity, pear [58]	>1	74.15	715.98	393.31 ^b^ ± 179.89	199.76	890.39	631.90 ^a^ ± 230.08	365.04	499.41	432.23 ^ab^ ± 54.86
Ethyl vanillate	3000 [63]	creamy, vanilla [61]		29.10	747.97	160.67 ^a^ ± 178.33	0.32	151.67	28.54 ^a^ ± 50.99	60.33	135.82	98.08 ^a^ ± 30.82
Ethyl-2-hydroxy-3-methylbutanoate				9.07	440.28	99.98 ^b^ ± 88.60	67.73	653.62	431.39 ^a^ ± 217.56	23.25	45.36	34.31 ^b^ ± 9.03
Ethyl-2-hydroxybutanoate				4.24	81.79	26.00 ^a^ ± 23.45	0.98	33.32	14.09 ^a^ ± 13.99	14.62	26.73	20.68 ^a^ ± 4.94
Ethyl-2-methylbutanoate	18 [54]	apple, strawberry [61]	>1	2.91	106.06	30.43 ^a^ ± 21.68	0.14	29.06	9.42 ^b^ ± 9.82	5.54	17.86	11.70 ^ab^ ± 5.03
Ethyl-2-oxopropanoate				38.2	253.61	126.68 ^a^ ± 71.24	0.14	217.10	55.96 ^a^ ± 96.89	64.31	116.59	90.45 ^a^ ± 21.34
Ethyl-3-ethoxypropanoate				0.02	2.64	0.54 ^a^ ± 0.68	0.05	0.14	0.13 ^a^ ± 0.46	0.02	0.10	0.06 ^a^ ± 0.03
Ethyl-3-hydroxybutanoate	20,000 [66]	grape, fruity, caramel, toasted [75]		0.11	75.28	21.75 ^a^ ± 28.47	0.25	1.61	0.93 ^b^ ± 10.12	0.96	76.04	38.50 ^a^ ± 30.65
Ethyl-3-hydroxyhexanoate	45 [58]	rubber [46]		0.20	12.35	7.19 ^a^ ± 3.04	0.26	10.43	4.99 ^a^ ± 2.96	7.33	7.91	7.62 ^a^ ± 0.24
Ethyl-3-methylbutanoate	3 [54]	fruity, pineapple [46]	>1	6.71	150.40	51.39 ^a^ ± 30.19	28.95	92.38	61.76 ^a^ ± 20.19	8.71	33.35	21.03 ^a^ ± 10.06
Ethyl-hydroxyhexanoate				0.15	376.69	120.86 ^a^ ± 92.70	0.50	161.62	20.64 ^b^ ± 69.54	68.68	92.95	80.82 ^ab^ ± 9.91
Ethylmethyl succinate				12.98	64.42	32.58 ^a^ ± 13.00	0.02	40.21	8.00 ^b^ ± 13.89	22.09	43.41	32.75 ^a^ ± 8.70
Hexyl acetate	670 [57]	fruity, green, sweet [61]		n.d.	183.29	51.91 ^a^ ± 60.85	0.13	161.50	56.90 ^a^ ± 7.88	49.15	83.69	66.42 ^a^ ± 14.10
Isoamyl acetate	30 [62]	banana [58]	>1	117.76	2825.79	436.95 ^a^ ± 552.20	76.89	726.31	473.72 ^a^ ± 240.34	599.36	610.28	604.82 ^a^ ± 4.46
Isoamyl lactate	1.6 [60]	fruity, apple, banana [46]	>1	0.17	602.50	144.88 ^ab^ ± 158.71	1.10	47.61	17.46 ^b^ ± 15.35	173.59	218.43	196.01 ^a^ ± 18.31
Isobutyl acetate	6140 [58]	apple, banana [61]		11.36	77.84	23.30 ^b^ ± 15.09	10.38	38.33	21.38 ^b^ ± 9.35	39.99	42.06	41.03 ^a^ ± 0.85
Isobutyl lactate	340,000 [58]			3.36	77.54	28.01 ^b^ ± 21.70	27.72	346.96	112.05 ^a^ ± 109.45	22.89	26.33	24.61 ^b^ ± 1.40
Methyl hexadecanoate	>2000 [74]			0.41	1.18	0.78 ^a^ ± 0.36	n.d.	1.61	0.84 ^a^ ± 0.54	-	-	n.d.
Methyl hydroxyisovalerate				2.14	323.55	52.30 ^a^ ± 69.63	0.67	57.49	18.95 ^a^ ± 22.50	4.92	50.53	27.73 ^a^ ± 18.62
Methyl hexanoate	84 [76]			n.d.	1.42	0.41 ^b^ ± 0.39	n.d.	0.75	0.43 ^b^ ± 0.24	1.5	1.84	1.67 ^a^ ± 0.14
Methyl octadecanoate				-	-	n.d.	-	-	n.d.	n.d.	1.04	0.52 ± 0.42
Methyl-2-furoate				0.05	1.24	0.56 ^b^ ± 0.36	0.09	24.54	5.00 ^a^ ± 7.36	0.52	0.90	0.71 ^b^ ± 0.16
Methyl-3-hydroxyoctanoate				0.06	0.47	0.17 ^b^ ± 0.10	0.10	29.40	10.08 ^a^ ± 9.45	0.11	0.18	0.15 ^b^ ± 0.03
Methyl geranoate				0.33	1.84	1.10 ^a^ ± 0.64	0.46	2.04	0.88 ^a^ ± 1.03	0.64	0.73	0.69 ^a^ ± 0.04
*o*-Methylbenzyl acetate				2.59	91.59	30.20 ^a^ ± 23.69	2.70	208.21	71.44 ^a^ ± 74.59	5.75	18.63	12.19 ^a^ ± 5.26
Phenyl acetate	250 [60]			0.25	789.13	87.35 ^a^ ± 226.77	0.15	2.03	1.25 ^a^ ± 1.72	0.11	0.36	0.24 ^a^ ± 0.10
Fatty acids												
2-Methylpropionic acid	230 [55]	rancid, cheesy [55]		0.92	9.11	3.31 ^b^ ± 2.06	4.82	16.96	8.67 ^a^ ± 4.22	3.94	4.53	4.24 ^b^ ± 0.24
Butanoic acid	400 [55]	rancid, cheese [51]		0.20	1.91	1.08 ^a^ ± 0.47	0.12	0.97	0.84 ^b^ ± 0.36	0.96	1.09	1.03 ^a^ ± 0.05
Decanoic acid	1000 [73]	rancid, waxy [51]	>1	851	5665	2456 ^a^ ± 1324	0.94	2100	798.88 ^b^ ± 1155.30	997.14	2434	1715.72 ^ab^ ± 586.71
Heptanoic acid	3000 [53]	rancid, cheesy [51]		0.96	2.19	1.39 ^b^ ± 0.35	0.11	13.87	5.65 ^a^ ± 4.18	1.13	1.85	1.49 ^b^ ± 0.29
Hexanoic acid	420 [53]	cheese, oily [58]	>1	137	10848	7006 ^a^ ± 2541	12.62	739.12	318.30 ^b^ ± 250.82	195.35	303.17	249.26 ^b^ ± 44.02
Isovaleric acid	33 [62]	sweat, rancid [51]	>1	0.78	43.93	7.01 ^ab^ ± 10.17	0.32	4.71	3.03 ^b^ ± 1.58	13.99	17.14	15.57 ^a^ ± 1.29
Octanoic acid	500 [54]	rancid, oily [61]	>1	2097	9123	6207 ^a^ ± 1937	4.81	6453	1252.90 ^b^ ± 2500.16	5700	7592	6646 ^a^ ± 772
Propanoic acid	8100 [57]	rancid, oily [46]		0.07	16.58	4.15 ^b^ ± 3.65	2.34	38.37	24.27 ^a^ ± 14.05	3.06	9.12	6.09 ^b^ ± 2.47
Other alcohols												
1,4-Butandiol				0.04	14.49	7.37 ^a^ ± 5.27	0.5	2.95	2.29 ^a^ ± 2.05	3.47	12.07	7.77 ^a^ ± 3.51
2,6-Dimethyl-4-heptanol				57.74	657.53	246.34 ^a^ ± 125.92	4.55	283.81	91.41 ^b^ ± 110.96	119.63	163.54	141.59 ^ab^ ± 17.93
2-Butoxy-ethanol				2.69	75.65	31.87 ^a^ ± 22.62	0.20	20.43	10.31 ^b^ ± 6.15	12.01	45.16	28.59 ^ab^ ± 13.53
2-Ethyl-1-hexanol				8.01	34.46	16.47 ^a^ ± 7.14	0.48	40.55	12.17 ^a^ ± 19.41	26.71	28.84	27.78 ^a^ ± 0.87
3,4-Dimethyl-2-hexanol				9.80	1579.33	169.17 ^a^ ± 396.65	0.56	71.53	29.10 ^a^ ± 25.57	98.14	135.69	116.92 ^a^ ± 15.33
3-Ethoxy-1-propanol	50,000 [58]	overripe, pear [75]		3.60	148.12	61.84 ^a^ ± 44.46	1.29	41.05	7.87 ^b^ ± 12.88	23.25	90.11	56.68 ^ab^ ± 27.30
3-Methyl-1-pentanol	50,000 [49]			73.67	225.57	137.92 ^a^ ± 38.41	55.52	113.69	89.29 ^b^ ± 22.35	87.19	94.30	90.75 ^b^ ± 2.90
3-Methyl-3-buten-1-ol				10.54	31.23	16.35 ^a^ ± 5.10	0.45	23.48	6.30 ^b^ ± 4.70	16.11	18.34	17.23 ^a^ ± 0.91
4-Ethylcyclohexanol				0.15	6.6	1.61 ^a^ ± 1.66	0.17	3.20	3.14 ^a^ ± 0.11	2.44	2.73	2.59 ^a^ ± 0.12
4-Methyl-1-pentanol	50,000 [58]	almond, toasted [46]		35.03	147.72	70.08 ^a^ ± 28.06	25.93	60.72	38.90 ^b^ ± 15.49	34.91	39.79	37.35 ^b^ ± 1.99
Benzylalcohol	10,000 [64]	roasted, toasted, sweet, fruity [51]		1.52	19.09	6.69 ^a^ ± 5.13	2.08	6.79	3.64 ^a^ ± 1.70	3.18	3.71	3.45 ^a^ ± 0.22
Cyclohexanol	160,000 [65]			0.03	4.22	1.39 ^a^ ± 1.40	0.20	6.24	2.30 ^a^ ± 2.25	0.15	3.35	1.75 ^a^ ± 1.31
Furfuril alkohol	15,000 [58]	sweet, nutty [61]		0.29	45.10	13.32 ^a^ ± 12.84	0.21	12.00	6.15 ^b^ ± 6.80	10.53	11.85	11.19 ^ab^ ± 0.54
2,3-Butanediol	668,000 [23]	buttery, creamy [58]		0.34	564.66	158.57 ^a^ ± 135.96	13.97	157.11	45.37 ^b^ ± 110.71	138.48	180.36	159.42 ^a^ ± 17.10
Sulfur compounds												
4-(Methylthio)-1-butanol	1000 [40]	metallic-bitter, garlic, earthy [46]		0.02	28.12	13.23 ^b^ ± 8.57	8.15	932.95	548.45 ^a^ ± 352.40	11.97	19.61	15.79 ^b^ ± 3.12
3-(Methylthio)-1-propanol	1000 [62]	cooked potato [61]		0.42	186.93	89.71 ^a^ ± 41.91	n.d.	123.66	23.61 ^b^ ± 17.69	18.26	45.94	32.10 ^b^ ± 11.30
Other compounds												
2,5-Hexadione				0.38	4.53	2.89 ^a^ ± 1.15	n.d.	1.07	0.97 ^b^ ± 0.76	2.45	2.69	2.57 ^a^ ± 0.10
2,7-Octanedione				0.11	81.61	11.43 ^a^ ± 16.45	n.d.	42.76	22.16 ^a^ ± 16.45	n.d.	3.48	1.74 ^a^ ± 1.42
2 H-Pyran-2,6(3H)-dione				0.19	48.76	24.08 ^a^ ± 18.89	n.d.	70.58	10.39 ^a^ ± 24.30	0.49	0.50	0.50 ^a^ ± 0.00
2-Pentylfuran	2000 [65]			3.78	9.73	6.63 ^b^ ± 1.80	3.87	14.25	9.81 ^a^ ± 2.78	8.48	8.94	8.71 ^ab^ ± 0.19
3-Penten-2-on				0.52	1021.26	79.69 ^a^ ± 215.90	n.d.	3.89	3.81 ^a^ ± 1.71	1.19	3.99	2.59 ^a^ ± 1.14
4-Hydroxy-4-methyl-2-pentanone				n.d.	14.52	5.85 ^a^ ± 2.79	n.d.	5.33	1.64 ^b^ ± 1.32	2.63	3.84	3.24 ^ab^ ± 0.49
4-Methyl-2-penten-2-one				n.d.	4.14	0.93 ^b^ ± 1.25	1.36	54.51	30.18 ^a^ ± 20.50	n.d.	0.32	0.16 ^b^ ± 0.13
5-Ethyl-4-methyl-3-heptanone				0.26	89.89	16.29 ^a^ ± 22.79	0.11	55.89	12.56 ^a^ ± 16.90	0.60	1.44	1.02 ^a^ ± 0.34
Acetoin	150,000 [54]	buttery, creamy [58]		17.30	2904.72	310.34 ^a^ ± 729.87	0.96	131.26	34.45 ^a^ ± 50.24	176.86	247.6	212.23 ^a^ ± 28.88
Acetylfurane				3.81	31.36	15.60 ^a^ ± 6.09	0.13	17.53	14.33 ^a^ ± 7.14	12.41	19.12	15.77 ^a^ ± 2.74
Benzofurane	350 [50]			2.60	178.34	34.30 ^a^ ± 38.71	0.31	22.68	5.32 ^b^ ± 7.13	8.64	20.46	14.55 ^ab^ ± 4.83
Dihydro-2-methyl-3(2H)-furanone				n.d.	8.48	2.55 ^a^ ± 1.95	0.21	9.79	3.75 ^a^ ± 2.81	1.12	1.50	1.31 ^a^ ± 0.16
N-(2-phenylethyl)acetamide				0.40	2.91	1.53 ^b^ ± 0.51	1.14	25.63	8.59 ^a^ ± 8.63	0.98	1.36	1.17 ^b^ ± 0.16
N-Ethylacetamide				15.33	56.90	31.59 ^a^ ± 11.28	2.41	52.00	19.32 ^a^ ± 17.69	30.24	40.38	35.31 ^a^ ± 4.14

ODT- odour detection treshold, OAV-odour active value, MIN-minimum value, MAX-maximum value, SD-standard deviation; Means mean± S.D. are calculated only from samples in which analytes were quantified; Means with different superscript letters in the same row differ significantly (*p* ≤ 0.05).

**Table 4:** Summary of the variables selection and ranking in Discriminant analysis using forward stepwise model for discrimination among sparkling wines samples from three vine growing region.

Rank	Variable Included	Partial R²	F	*p*	Wilks’ Lambda ^a^	*p*
1	Methyl hexanoate	0.86	88.5	<0.1 × 10^−4^	1.45 × 10^−1^	<0.1 × 10^−4^
2	Hexanoic acid	0.81	60.0	<0.1 × 10^−4^	2.82 × 10^−2^	<0.1 × 10^−4^
3	4-Hydroxy-4-methyl-2-pentanone	0.48	13.0	1.02 × 10^−4^	1.46 × 10^−2^	<0.1 × 10^−4^
4	Heptanal	0.57	17.9	<0.1 × 10^−4^	6.29 × 10^−3^	<0.1 × 10^−4^
5	Octanoic acid	0.58	17.7	<0.1 × 10^−4^	2.66 × 10^−3^	<0.1 × 10^−4^
6	3-Penten-2-on	0.42	8.9	1.21 × 10^−3^	1.55 × 10^−3^	<0.1 × 10^−4^
7	Isobutyl acetate	0.40	7.8	2.41 × 10^−3^	9.41 × 10^−4^	<0.1 × 10^−4^
8	2-Methylpropionic acid	0.43	8.8	1.47 × 10^−3^	5.34 × 10^−4^	<0.1 × 10^−4^
9	1,4-Butandiol	0.40	7.4	3.40 × 10^−3^	3.18 × 10^−4^	<0.1 × 10^−4^
10	Acetylfurane	0.42	7.5	3.43 × 10^−3^	1.85 × 10^−4^	<0.1 × 10^−4^
11	Isobutyl lactate	0.41	6.8	5.54 × 10^−3^	1.10 × 10^−4^	<0.1 × 10^−4^
12	Ethyl linoleate	0.58	13.0	2.78 × 10^−4^	4.66 × 10^−5^	<0.1 × 10^−4^
13	Propanoic acid	0.46	7.8	3.63 × 10^−3^	2.50 × 10^−5^	<0.1 × 10^−4^
14	2H-Pyran-2,6(3H)-dione	0.53	9.4	1.79 × 10^−3^	1.19 × 10^−5^	<0.1 × 10^−4^
15	TDN	0.52	8.6	2.86 × 10^−3^	5.70 × 10^−6^	<0.1 × 10^−4^
16	Ethyl linalyl acetal	0.48	7.0	7.30 × 10^−3^	2.96 × 10^−6^	<0.1 × 10^−4^
17	Ethyl-2-oxopropanoate	0.60	10.7	1.53 × 10^−3^	1.17 × 10^−6^	<0.1 × 10^−4^
18	Benzaacetaldehyde	0.51	6.7	9.94 × 10^−3^	5.76 × 10^−7^	<0.1 × 10^−4^
19	Hexanal	0.51	6.2	1.42 × 10^−3^	2.84 × 10^−7^	<0.1 × 10^−4^
20	β-Farnesen	0.42	4.0	4.91 × 10^−2^	1.64 × 10^−7^	<0.1 × 10^−4^
21	Benzaldehyde	0.58	6.9	1.33 × 10^−2^	6.92 × 10^−8^	<0.1 × 10^−4^

^a^ Wilks’ Lambda test of the assumption of equality of the mean vectors of classes.

**Table 5:** Fisher distance among samples of sparkling wines from three vine-growing regions based on 3 volatile compounds.

	Krašić	Plešivica	Zelina
Krašić	0	26.3 ***	14.0 ***
Plešivica			57.3 ***
Zelina			0

*p*-values for Fisher distances: *** *p* < 0.001.

**Table 6:** Odour activity values (OAV) and relative odour contribution (ROC) in sparkling wines.

Parameters	Vine-Growing Regions					
	Plašivica (*n* = 19)		Zelina (*n* = 8)		Krašić (*n* = 3)	
	OAV	ROC(%)	OAV	ROC(%)	OAV	ROC(%)
Aldehydes						
2,4-Nonadienal	18.88	5.1	7.77	2.25	21	6.08
2-Heptenal	46.76	14.25	3.71	1.15	33.13	9.61
Decanal	12.85	3.5	27.61	8.28	13	3.76
Hexanal	40.73	11.06	86.74	26.38	27.33	7.92
Nonanal	10.04	2.7	3.65	1.11	17.47	5.06
∑		36.61		39.17		32.43
Higher alcohols						
1-Hexanol	0.74	0.2	0.64	0.19	1.18	0.34
Isoamyl alcohol	0.79	0.21	1.32	0.4	0.68	0.19
Phenyethyl alcohol	0.69	0.18	1.12	0.34	0.57	0.16
∑		0.59		0.93		0.69
Volatile phenols						
4-Vinylguaicol	2.85	0.77	0.71	0.21	6.4	1.85
Terpenes						
Geraniol	0.11	0.02	4.4	1.34	0.39	0.11
Hotrienol	0.28	0.07	0.26	0.07	1.8	0.52
Linalool	0.45	0.12	1.8	0.55	2.92	0.84
∑		0.21		1.96		1.47
C13-norisoprenoides						
*β*-Damascenone	38.2	10.4	73.6	22.57	2.8	0.81
TDN	1.27	0.3	1.94	0.59	0.27	0.07
∑		10.7		23.16		0.88
Lactones						
*γ*-Nonalacton	0.3	0.08	1	0.31	0.45	0.13
Esters						
Ethyl butanoate	13.5	3.4	20.85	6.39	18.1	5.24
Ethyl hexanoate	23.85	6.39	37.14	11.54	31.07	9.16
Ethyl octanoate	0.67	0.18	1.08	0.33	0.74	0.21
Ethyl-2-methylbutanoate	1.69	0.46	0.52	0.15	0.65	0.18
Ethyl-3-methylbutanoate	17.13	4.66	20.33	6.43	6.67	1.93
Isoamyl aceatet	14.53	2.95	15.76	4.83	20.13	5.83
Isoamyl lactate	90	24.52	10.9	3.34	122.5	35.36
∑		42.56		33.01		57.91
Fatty acids						
Decanoic acid	2.4	0.62	0.79	0.24	1.7	0.49
Hexanoic acid	16.66	4.43	0.75	0.23	0.59	0.17
Isovaleric acid	0.21	0.05	0.09	0.02	0.45	0.13
Octanoic acid	12.41	3.38	2.5	0.76	13.29	3.85
∑		8.48		1.25		4.64

**Table 7:** Identification and quantification parameters for GC-MS analysis.

Parameters	RT/min	LRI	Q_ion_	ID	Chemical Standard
Aldehydes					
2,4-Decadienal	67.87	1837	81	S, MS, RI	Sigma
2,4-Heptadienal *(E,E)*	49,36	1518	81	S, MS, RI	Sigma
2,4-Heptadienal *(Z,Z)*	47.49	1488	81	S, MS, RI	Sigma
2,4-Nonadienal	61.83	1728	81	S, MS, RI	Sigma
2-Heptenal	38.01	1344	41	S, MS, RI	Sigma
5-Hydroxymethylfurfural	100.53	2525	97	S, MS, RI	Sigma
5-Methyl-furfural	54.47	1601	110	S, MS, RI	Sigma
Benzeneacetaldehyde	58.90	1676	91	S, MS, RI	Sigma
Benzaldehyde	51.54	1553	106	S, MS, RI	Sigma
Decanal	49.32	1518	43	S, MS, RI	Sigma
Furfural	47.70	1492	96	S, MS, RI	Sigma
Heptanal	28.29	1201	44	S, MS, RI	Sigma
Hexanal	21.27	1097	44	S, MS, RI	Sigma
Nonanal	42.53	1412	57	S, MS, RI	Sigma
Higher alcohols					
1-Butanol	25.20	1555	56	S, MS, RI	Sigma
1-Decanol	64.67	1779	55	S, MS, RI	Sigma
1-Heptanol	46.33	1471	70	S, MS, RI	Sigma
1-Hexanol	39.61	1368	41	S, MS, RI	Sigma
1-Methoxy-2-propanol	24.03	1138	45	S, MS, RI	Sigma
1-Octadecanol	104.93	2604	83	S, MS, RI	Sigma
1-Octanol	52.77	1573	56	S, MS, RI	Sigma
1-Pentanol	32.50	1263	42	S, MS, RI	Sigma
2-Heptanol	37.20	1332	45	S, MS, RI	Sigma
*trans*-2-Hexene-1-ol	43.28	1423	57	S, MS, RI	Sigma
*cis*-2-Hexene-1-ol	41.76	1446	57	S, MS, RI	Sigma
2-Methyl-1-butanol	29.55	1220	57	S, MS, RI	Sigma
2-Pentadecanol	75.47	1983	45	S, MS, RI	Sigma
2-Pentene-1-ol	37.40	1335	57	S, MS, RI	Sigma
*cis*-3-Hexene-1-ol	41.76	1405	41	S, MS, RI	Sigma
*trans*-3-Hexene-1-ol	40.42	1380	41	S, MS, RI	Sigma
3-Octanol	42.19	1407	59	S, MS, RI	Sigma
Isoamyl alcohol	29.80	1223	55	S, MS, RI	Sigma
Isobutanol	21.66	1102	43	S, MS, RI	Sigma
Phenylethyl alcohol	71.53	1907	91	S, MS, RI	Sigma
Volatile phenols					
4-Ethylguaiacol	79.53	2066	85	S, MS, RI	Sigma
4-Ethylphenol	86.58	2216	107	S, MS, RI	Sigma
4-Vinylguaiacol	87.46	2236	150	S, MS, RI	Sigma
4-Vinylphenol	96.41	2439	120	S, MS, RI.	Sigma
Eugenol	86.07	2205	164	S, MS, RI	Sigma
Guaiacol	70.93	1895	124	S, MS, RI	Sigma
Homovanillyl alcohol	116.11	2817	137	- S, MS, RI	Sigma
Vanillin	103.41	2577	151	S, MS, RI	Sigma
Tyrosol	97.96	2473	107	S, MS, RI	Sigma
Terpenes					
1,8-Terpin	82.19	2122	81	S, MS, RI	Sigma
6,7-Dihydro-7-hydroxylinalool	75.92	1992	71	S, MS, RI	Boc Science
8-Hidroxylinalool	92.01	2339	43	S, MS, RI	Aurora Fine Chemicals
*α*-Terpineol	61.39	1720	59	S, MS, RI	Extrasynthese
*β*-Farnesen	59.30	1683	69	S, MS, RI	Extrasynthese
*β*-Ocimene	62.74	1744	93	S, MS, RI	Extrasynthese
*cis*-Linalool oxide, furan	47.54	1489	59	S, MS, RI	Extrasynthese
Citronelol	65.03	1785	69	S, MS, RI	Extrasynthese
*δ*-Carene	37.71	1340	93	S, MS, RI	Extrasynthese
Dihydroactinidiolide	94.54	2398	111	S, MS, RI	Boc Science
Farnesol	93.73	2380	69	S, MS, RI	Extrasynthese
*γ*-Terpinene	32.29	1260	93	S, MS, RI	Extrasynthese
Geraniol	69.49	1868	69	S, MS, RI	Extrasynthese
Geranyl acetate	91.81	2334	69	S, MS, RI	Boc Science
Hotrienol	56.14	1629	71	S, MS, RI	Boc Science
Linalool	52.17	1563	71	S, MS, RI	Extrasynthese
Linalool oxide, pyran	63.76	1762	68	S, MS, RI	Boc Science
Neric acid	93.83	2381	69	S, MS, RI	Extrasynthese
Nerol	67.04	1822	69	S, MS, RI	Extrasynthese
Nerolidol	79.20	2059	69	S, MS, RI	Boc Science
Pseudoionon	83.10	2141	58	S, MS, RI	Boc Science
Terpendiol I	62.31	1736	82	MS	
Terpendiol II	74.54	1965	67	MS	
Terpinene-4-ol	55.87	1624	71	S, MS, RI	Extrasynthese
Tetrahydrolinalool	44.62	1444	73	S, MS, RI	Boc Science
*trans*-Linalool oxide, furan	45.67	1460	59	S, MS, RI	Extrasynthese
*trans*-Rose oxide	39.64	1368	139	S, MS, RI	Sigma
Linalyl formate	47.38	1487	69	S, MS, RI	Boc Science
Ethyl linalyl acetal	46.95	1480	73	S, MS, RI	Boc Science
2,6-Dimethyl-3,7-octadiene-2,6-diol	61.25	1718	82	S, MS, RI	Boc Science
2,6-Dimethyl-7-octene-2,6-diol	76.07	1995	71	S, MS, RI	Boc Science
Menthol	57.94	1660	71	S, MS, RI	Sigma
Ocimenol	58.50	1669	93	S, MS, RI	Extrasynthese
C13- norisoprenoids					
*α*-Ionol	72.23	1920	95	S, MS, RI	Sigma
*α*-Ionon	70.00	1877	121	S, MS, RI	Sigma
*β*-Damascenone	68.46	1848	69	S, MS, RI	Sigma
TDN	64.43	1774	157	S, MS, RI	Boc Science
Lactones					
*cis*-Whiskey lactone	75.95	1993	91	S, MS, RI	Sigma
*δ*-Nonalactone	87.43	2235	99	S, MS, RI	Sigma
*γ*-Butyrolactone	58.30	1666	42	S, MS, RI	Sigma
*γ*-Decalactone	85.06	2183	85	S, MS, RI	Sigma
*γ*-Hexalactone	62.40	1738	85	S, MS, RI	Sigma
*γ*-Nonalacton	79.55	2067	85	S, MS, RI	Sigma
*γ*-Octalacton	73.94	1953	85	S, MS, RI	Sigma
*γ*-Undecalactone	90.28	2299	85	S, MS, RI	Sigma
*trans*-Whiskey lactone	72.28	1921	99	S, MS, RI	Sigma
Esters					
2-Phenylethyl acetate	68.28	1845	105	S, MS, RI	Sigma
Diethyl glutarate	65.93	1801	143	S, MS, RI	Sigma
Methyl hydroxyisovalerate	70.10	1879	131	S, MS, RI	Sigma
Diethyl hydroxysuccinate	97.00	2454	101	S, MS, RI	Boc Science
Diethyl malonate	54.35	1598	115	S, MS, RI	Boc Science
Diethyl oxalate	43.67	1429	59	S, MS, RI	Sigma
Diethyl succinate	60.09	1697	101	S, MS, RI	Sigma
Dimethyl malate	78.27	2040	103	S, MS, RI	Sigma
Ethyl benzeneacetate	66.64	1814	91	S, MS, RI	Sigma
Ethyl butanoate	18.32	1053	71	S, MS, RI	Sigma
Ethyl decanoate	57.48	1652	88	S, MS, RI	Sigma
Ethyl furoate	57.28	1648	95	S, MS, RI	Sigma
Ethyl hexadecanoate	88.91	2268	88	S, MS, RI	Sigma
Ethyl hexanoate	31.40	1247	88	S, MS, RI	Sigma
Ethyl hydrogensuccinate	96.70	2445	101	S, MS, RI	Sigma
Ethyl lactate	39.28	1363	45	S, MS, RI	Sigma
Ethyl linoleate	101.66	2645	67	S, MS, RI	Sigma
Ethyl octanoate	45.05	1447	88	S, MS, RI	Sigma
Ethyl vanillate	107.17	1425	151	S, MS, RI	Sigma
Ethyl-2-hydroxy-3-methylbutanoate	44.82	1067	73	S, MS, RI	Sigma
Ethyl-2-hydroxybutanoate	43.41	1290	59	S, MS, RI	Boc Science
Ethyl-2-methylbutanoate	19.29	1350	57	S, MS, RI	Boc Science
Ethyl-2-oxopropanoate	34.34	1541	43	S, MS, RI	Sigma
Ethyl-3-ethoxypropanoate	38.39	1350	59	S, MS, RI	Boc Science
Ethyl-3-hydroxybutanoate	50.79	1541	43	S, MS, RI	Boc Science
Ethyl-3-hydroxyhexanoate	72.00	1915	117	S, MS, RI	Boc Science
Ethyl-3-methylbutanoate	20.30	1082	88	S, MS, RI	Sigma
Ethyl-hydroxyhexanoate	52.19	1563	69	S, MS, RI	Sigma
Ethylmethyl succinate	57.80	1657	115	S, MS, RI	Mol Port
Hexyl acetate	34.16	1287	43	S, MS, RI	Sigma
Isoamyl acetate	23.81	1135	43	S, MS, RI	Sigma
Isoamyl lactate	53.70	1588	45	S, MS, RI	Sigma
Isobutyl acetate	16.97	1032	43	S, MS, RI	Sigma
Isobutyl lactate	46.90	1479	45	S, MS, RI	Sigma
Methyl hexadecanoate	87.32	2233	74	S, MS, RI	Sigma
Methyl hexanoate	28.33	1202	74	S, MS, RI	Sigma
Methyl octadecanoate	96.36	2438	74	S, MS, RI	Sigma
Methyl-2-furoate	54.67	1603	95	S, MS, RI	Sigma
Methyl-3-hydroxyoctanoate	77.70	2029	103	S, MS, RI	Boc Science
Methyl geranoate	61.25	1718	69	S, MS, RI	Boc Science
*o*-Methylbenzyl acetate	92.95	2361	104	S, MS, RI	Sigma
Phenyl acetate	57.31	1649	94	S, MS, RI	Sigma
Fatty acids					
2-Methylpropionic acid	54.73	1604	43	S, MS, RI	MolPort
Butanoic acid	58,56	1671	60	S, MS, RI	Sigma
Decanoic acid	90.90	2313	60	S, MS, RI	Sigma
Heptanoic acid	76.17	1997	60	S, MS, RI	Sigma
Hexanoic acid	70.25	1882	60	S, MS, RI	Sigma
Isovaleric acid	60.88	1711	60	S, MS, RI	Sigma
Octanoic acid	81.05	2097	60	S, MS, RI	Sigma
Propanoic acid	53.16	1579	74	S, MS, RI	Sigma
Other alcohols					
1,4-Butandiol	73.70	1949	42	S, MS, RI	Sigma
2,6-Dimethyl-4-heptanol	52.26	1565	69	S, MS, RI	Sigma
2-Butoxy-ethanol	41.34	1394	57	S, MS, RI	Sigma
2-Ethyl-1-hexanol	48.54	1505	57	S, MS, RI	Sigma
3,4-Dimethyl-2-hexanol	35.48	1306	45	S, MS, RI	Sigma
3-Ethoxy-1-propanol	41.34	1394	59	S, MS, RI	Sigma
3-Methyl-1-pentanol	37.86	1342	56	S, MS, RI	Sigma
3-Methyl-3-buten-1-ol	32.63	1265	41	S, MS, RI	Sigma
4-Ethylcyclohexanol	49.37	1518	81	S, MS, RI	Sigma
4-Methyl-1-pentanol	37.00	1329	56	S, MS, RI	Sigma
Benzylalcohol	71.50	1906	78	S, MS, RI	Sigma
Cyclohexanol	41.95	1403	43	S, MS, RI	Sigma
Furfuril alkohol	59.56	1688	98	S, MS, RI	Sigma
2,3-Butanediol	51.90	1559	45	S, MS, RI	Sigma
Sulfur compounds					
4-(Methylthio)-1-butanol	69.56	1869	61	S, MS, RI	Sigma
3-(Methylthio)-1-propanol	62.80	1745	106	S, MS, RI	Sigma
Other compounds					
2,5-Hexadione	50.09	1530	43	S, MS, RI	Sigma
2,7-Octanedione	41.95	1403	43	S, MS, RI	Aurora Fine Chemicals
2H-Pyran-2,6(3H)-dione	78.28	2040	112	S, MS, RI	MolPort
2-Pentylfuran	31.40	1247	81	S, MS, RI	Sigma
3-Penten-2-on	24.21	1141	69	S, MS, RI	Sigma
4-Hydroxy-4-methyl-2-pentanone	40.46	1381	43	S, MS, RI	Sigma
4-Methyl-2-penten-2-one	24.65	1147	55	S, MS, RI	Sigma
5-Ethyl-4-methyl-3-heptanone	26.19	1170	57	S, MS, RI	Aurora Fine Chemicals
Acetoin	35.55	1307	45	S, MS, RI	Sigma
Acetylfurane	50.27	1533	95	S, MS, RI	Sigma
Benzofurane	66.16	1805	118	S, MS, RI	Sigma
Dihydro-2-methyl-3(2*H*)-furanone	63.07	1750	43	S, MS, RI	Sigma
*N*-(2-phenylethyl)acetamide	106.27	2629	104	S, MS, RI	Sigma
*N*-Ethylacetamide	57.77	1657	43	S, MS, RI	Sigma

RT-retention time; LRI-linear retention indices; Q_ion_-ion qualifier; ID: S-retention time and mass spectrum consistent with standard, RI—retention index consistent with those find in the literature, MS—mass spectra consistent with those find in NIST02 electronic library.
